# Nonrestorative Sleep and Poor Self-Reported Physical and Mental Health among Middle-Aged Japanese Adults: A Cross-Sectional Study

**DOI:** 10.31662/jmaj.2025-0464

**Published:** 2026-05-15

**Authors:** Akiko Morimoto, Hideaki Furuki, Liqian Sun, Aina Muta, Nao Sonoda

**Affiliations:** 1 Graduate School of Nursing, Osaka Metropolitan University, Osaka, Japan

**Keywords:** nonrestorative sleep, self-reported health, physical health, mental health, middle-aged adults

## Abstract

**Introduction:**

Self-reported health is a robust indicator linked to mortality, morbidity, and health-related quality of life. Nonrestorative sleep―subjective unrefreshing sleep―is increasingly reported in middle-aged adults and is related to cardiometabolic and mental conditions, yet its relationship with perceived health in this group is underexplored. This study investigated the associations between nonrestorative sleep and poor self-reported physical and mental health among middle-aged adults in Japan.

**Methods:**

A cross-sectional postal survey was conducted in 2020 using a self-administered questionnaire among 33,902 community residents aged 40-64 years in five cities of Osaka Prefecture, Japan. After excluding missing data, 11,086 participants remained for analysis. Physical and mental health were assessed with standardized rating items, dichotomized into “very good/good/average” versus “poor/very poor.” Nonrestorative sleep was defined by the question, “Do you feel refreshed after a night’s sleep?” with “no” indicating nonrestorative sleep.

**Results:**

Among the study cohort, nonrestorative sleep was associated with increased risk ratios (RRs) for poor physical health (adjusted RR: 2.13; 95% confidence interval [CI]: 1.97-2.29) and poor mental health (adjusted RR: 2.21; 95% CI: 2.06-2.36). For participants without a history of present illness, the adjusted RRs were 2.55 (95% CI: 2.11-3.07) for poor physical health and 2.51 (95% CI: 2.18-2.89) for poor mental health. All models were adjusted for sociodemographic, behavioral, and health-related factors. Nonrestorative sleep consistently showed the highest RRs in the primary multivariable models.

**Conclusions:**

This large-scale study demonstrates that nonrestorative sleep is strongly associated with poor self-reported physical and mental health in middle-aged adults.

## Introduction

Self-reported health is a widely recognized indicator of individuals’ perceived overall health. Despite its simplicity, this measure has been consistently linked to objective outcomes such as mortality, morbidity, and health-related quality of life ^(1), (2), (3), (4), (5), (6), (7), (8)^. Middle adulthood (aged 40-64 years) is a period characterized by heightened vulnerability to chronic illness and psychosocial stress. Poor self-reported health during this stage has been associated with an increased risk of premature mortality, cardiovascular disease, diabetes, and depression ^(9), (10), (11)^, as well as subsequent cognitive and functional decline ^(12)^. Identifying modifiable contributors to health perception is therefore essential for nursing interventions aimed at strengthening well-being and preventing long-term deterioration.

Sleep quality has emerged as an important factor in this context. Nonrestorative sleep―commonly defined as the subjective experience of unrefreshing sleep―has been strongly associated with hypertension, diabetes, and depression ^(13), (14), (15), (16)^. National Health and Nutrition Survey data from Japan indicate increasing sleep complaints among middle-aged adults, with nonrestorative sleep frequently reported ^(17), (18)^. However, the relationship between nonrestorative sleep and self-reported health remains underexplored, particularly in this demographic, which faces rising sleep disturbances and chronic disease burden. While prior research has emphasized objective outcomes, fewer studies have addressed perceived health status―a gap with critical implications for nursing assessment and preventive care.

Therefore, this study examines cross-sectional associations between nonrestorative sleep and poor self-reported physical and mental health among middle-aged adults in Japan.

## Materials and Methods

### Study design and participants

A cross-sectional study was conducted in 2020 using a self-administered postal questionnaire. Participants received the questionnaire by mail, completed it independently, and returned it in a prepaid envelope. Completion time was estimated at 10 minutes, with a one-month return period. To increase responses, a reminder postcard was sent one week after the initial mailing. Full study details have been reported elsewhere ^(19)^.

The convenience sample included 33,902 community residents aged 40-64 years from five cities in Osaka Prefecture, Japan. Of these, 12,446 (36.7%) returned the questionnaire. After excluding 1,360 with missing data, 11,086 participants were included in the final analysis.

### Measurements

#### Self-reported physical and mental health

Self-reported health is a well-established and reliable indicator of health status ^(2)^ and has been widely applied in population-level and clinical studies ^(1), (3), (4), (5), (6), (7), (8)^. This indicator is based on an individual’s self-assessed rating of their health and is typically measured with a five-point Likert scale ^(20), (21), (22)^.

Self-reported physical health was assessed with the item: “How would you rate your current physical health?” Mental health was assessed with the item: “How would you rate your current mental health?” Both used the response options: “very good,” “good,” “average,” “poor,” and “very poor.” For analysis, responses were dichotomized into “very good,” “good,” and “average” vs. “poor” and “very poor” to identify participants with worse perceived health. Because the “very poor” category was sparse in our sample, we combined “poor” and “very poor” to avoid small cell counts and improve estimate stability.

#### Nonrestorative sleep

Nonrestorative sleep was evaluated with the item: “Do you feel refreshed after a night’s sleep?” Responses of “no” were classified as nonrestorative sleep, and responses of “yes” as restorative sleep ^(23)^. This definition aligns with the widely used description of nonrestorative sleep as the subjective experience of unrefreshing sleep ^(14)^.

#### Potential confounding factors

Data were collected on potential confounders including age, sex (male or female), living alone (yes or no), occupation (presence or absence), economic status (very good, good, average, poor, or very poor), education (9-12 years or >12 years), health literacy, smoking status (non-smoker, former smoker, or current smoker), alcohol consumption (non-drinker, occasional drinker, or daily drinker), physical activity (presence or absence), obesity (presence or absence), and number of visits to medical institutions per year.

Health literacy was assessed using the Communicative and Critical Health Literacy (CCHL) scale ^(24)^. Scores from five items were summed and divided by the number of items on the scale to yield a scale score (theoretical range, 1-5) ^(24)^. The reliability and validity of the CCHL have been confirmed ^(24)^, and Cronbach’s alpha in this study was 0.88. Physical activity was defined as ≥150 minutes of moderate-intensity physical activity per week ^(25), (26)^. Body mass index (BMI) was calculated as weight (kg)/height (m)^2^, using self-reported values. Obesity was defined as BMI ≥25.0 kg/m^2^ ^(27)^.

### Statistical analysis

We conducted two primary analyses: one in the entire sample, and the second in participants without regular visits to medical institutions (i.e., no history of present illness). Group differences between restorative and nonrestorative sleep were evaluated using t-tests for normally distributed continuous variables, Mann-Whitney U tests for non-normally distributed variables, and chi-squared tests for categorical variables.

Differences in the proportion of participants reporting poor physical and mental health across sleep groups were examined using chi-squared tests. Modified Poisson regression models were used to estimate multivariable-adjusted risk ratios (RRs) and 95% confidence intervals (CIs) for poor self-reported physical and mental health (response variable: 1 = poor/very poor; 0 = very good/good/average) associated with nonrestorative sleep ^(28)^. Covariates included age, sex, living arrangement, occupation, economic status, education, health literacy (CCHL score), smoking, alcohol use, physical activity, obesity, and medical visits per year. To assess robustness, we additionally performed sensitivity analyses stratified by regular-visit category: regular visits to non-psychiatric medical institutions only, psychiatric/psychosomatic institutions only, and both. Primary and stratified sensitivity analyses were performed as complete-case analyses for transparency and ease of interpretation. As an additional sensitivity analysis for missing data, we performed multiple imputation by chained equations ^(29)^. We generated 20 imputed datasets and combined estimates using Rubin’s rules, applying the same modified Poisson regression models. The imputation model included all variables in the analytic models (exposure, outcomes, and covariates) as well as auxiliary variables such as daily time use (working, housework, childcare, and caregiving hours) and health information sources (internet, healthcare providers, and family/friends/coworkers).

Analyses were performed using SPSS version 26 (IBM SPSS Japan, Tokyo, Japan) or R version 4.4.2 (R Foundation for Statistical Computing, Vienna, Austria). All *p*-values were two-tailed, with statistical significance set at <0.05 (alpha level = 0.05).

### Ethical considerations

The study protocol was written in accordance with the Declaration of Helsinki and was approved by the Institutional Review Boards of Osaka Prefecture University (date of approval 5 October 2020; approval no. 2020-28).

## Results

### Differences in characteristics between the restorative sleep and nonrestorative sleep groups

Table 1 summarizes participant characteristics by restorative and nonrestorative sleep status among middle-aged adults in Japan. Among the 11,086 participants in the entire sample, 4,559 (36.6%) reported nonrestorative sleep. In this sample, age (p < 0.001), sex (p < 0.001), occupation (p < 0.001), economic status (p < 0.001), education level (p < 0.001), health literacy (p < 0.001), smoking (p = 0.032), alcohol consumption (p = 0.049), physical inactivity (p < 0.001), obesity (p = 0.012), and annual medical visits (p < 0.001) differed significantly between restorative and nonrestorative sleep groups.

**Table 1.** Differences in Characteristics between Restorative Sleep and Nonrestorative Sleep Groups among Middle-Aged Adults in Japan.

Factors	Entire cohort (n = 11,086)			Those without a history of present illness (n = 3,658)		
	Restorative sleep	Nonrestorative sleep	p-Value	Restorative sleep	Nonrestorative sleep	p-Value
n	6,527	4,559		2,181	1,477	
Age (years)	54.8 (6.9)	54.3 (6.6)	<0.001	53.4 (6.8)	53.0 (6.6)	0.095
Female, %	58.9	62.1	<0.001	53.0	55.0	0.224
Living alone: Yes, %	14.0	14.2	0.814	12.6	14.5	0.101
Occupation: Absence, %	20.0	16.5	<0.001	15.5	12.0	0.002
Economic status: Poor and very poor, %	50.0	66.4	<0.001	52.6	69.2	<0.001
Education level: 9-12 years, %	49.4	53.5	<0.001	50.5	56.5	<0.001
Health literacy^*^ (points)	3.5 (0.8)	3.4 (0.8)	<0.001	3.6 (0.7)	3.4 (0.8)	<0.001
Smoking: Current smoker, %	20.9	22.5	0.032	25.6	28.4	0.060
Alcohol consumption: Daily drinker, %	24.9	23.2	0.049	27.7	28.1	0.813
Physical activity: Absence, %	65.8	77.6	<0.001	65.4	76.4	<0.001
Obesity: Presence, %	26.8	29.0	0.012	22.9	25.5	0.073
Annual visits to medical institutions (times/year)	5.0 (1.0, 12.0)	5.0 (2.0, 12.0)	<0.001	1.0 (0.0, 2.0)	1.0 (0.0, 2.5)	0.001

Age and health literacy were analyzed using t-tests and are shown as mean (standard deviation). Annual visits to medical institutions were analyzed with Mann-Whitney U tests and are shown as median (25th, 75th percentiles). Dichotomous and categorical data were analyzed with chi-squared tests and are presented as %. ^*^Communicative and Critical Health Literacy scale score.

Among the 3,658 participants without a history of present illness, 1,477 (40.4%) reported nonrestorative sleep. In this group, significant differences were observed for occupation (p = 0.002), economic status (p < 0.001), education (p < 0.001), health literacy (p < 0.001), physical inactivity (p < 0.001), and annual medical visits (p = 0.001). Stratified analyses by regular-visit category (regular visits to non-psychiatric medical institutions only, psychiatric/psychosomatic institutions only, or both) are presented in Table S1. The numbers of participants with nonrestorative sleep were 2,730, 108, and 244 in the non-psychiatric-only, psychiatric/psychosomatic-only, and both-visit categories, respectively.

### Associations between nonrestorative sleep and poor self-reported physical and mental health

Table 2 shows self-reported health differences by sleep status. In both subgroups―the entire cohort and those without a history of present illness―the proportions of participants reporting poor physical and mental health were significantly higher in the nonrestorative sleep group than in the restorative sleep group. This pattern was also consistent across regular-visit categories (Table S2).

**Table 2.** Differences in Self-Reported Physical and Mental Health between Restorative Sleep and Nonrestorative Sleep Groups among Middle-Aged Adults in Japan.

Factors	Entire cohort (n = 11,086)			Those without a history of present illness (n = 3,658)		
	Restorative sleep	Nonrestorative sleep	p-Value	Restorative sleep	Nonrestorative sleep	p-Value
n	6,527	4,559		2,181	1,477	
Self-reported physical health, %			<0.001			<0.001
Very good	17.3	4.2		24.9	6.2	
Good	26.8	14.7		29.5	17.9	
Average	42.7	49.4		39.0	55.5	
Poor	11.2	26.1		6.1	17.5	
Very poor	2.1	5.6		0.6	2.8	
Poor self-reported physical health^*^, %	13.3	31.7	<0.001	6.7	20.3	<0.001
Self-reported mental health, %			<0.001			<0.001
Very good	16.9	5.0		19.9	6.4	
Good	25.4	13.0		27.9	15.0	
Average	42.8	44.1		41.0	45.9	
Poor	12.4	29.1		9.6	26.6	
Very poor	2.5	8.8		1.6	6.1	
Poor self-reported mental health^*^, %	14.9	37.9	<0.001	11.2	32.7	<0.001

Categorical data were analyzed with chi-squared tests and shown as %. Since percentages are rounded, the sum of the displayed percentages may not equal the displayed total for the corresponding category. ^*^Responses were dichotomized into: “very good/good/average” vs. “poor/very poor”.

Table 3 presents multivariable-adjusted RRs and 95% CIs for poor self-reported physical health associated with nonrestorative sleep. Among the entire cohort, after adjusting for age, sex, living alone, occupation, economic status, education, health literacy, smoking, alcohol consumption, physical activity, obesity, and annual medical visits, the adjusted RR for poor physical health was 2.13 (95% CI: 1.97-2.29; p < 0.001) in the nonrestorative sleep group compared with the restorative sleep group. Among participants without a history of present illness, the adjusted RR was 2.55 (95% CI: 2.11-3.07; p < 0.001). In both subgroups, nonrestorative sleep exhibited the highest RRs among all covariates, underscoring its strong independent association with poor physical health. Moreover, in the stratified models by regular-visit category, nonrestorative sleep remained associated with poor physical health across all categories (Table S3).

**Table 3.** Multivariable-Adjusted RRs and 95% CIs for Poor Self-Reported Physical Health due to Nonrestorative Sleep among Middle-Aged Adults in Japan.

Factors	Comparison	Entire cohort (n = 11,086)		Those without a history of present illness (n = 3,658)	
		Multivariable-adjusted RR (95% CI) for poor self-reported physical health	p-Value	Multivariable-adjusted RR (95% CI) for poor self-reported physical health	p-Value
Nonrestorative sleep	vs. Restorative sleep	2.13 (1.97-2.29)	<0.001	2.55 (2.11-3.07)	<0.001
Age	per 1 year	1.001 (0.996-1.007)	0.603	0.99 (0.98-1.01)	0.314
Female	vs. Male	0.98 (0.91-1.05)	0.566	0.87 (0.73-1.05)	0.156
Living alone: Yes	vs. No	1.13 (1.03-1.23)	0.009	1.10 (0.87-1.39)	0.419
Occupation: Absence	vs. Presence	1.73 (1.61-1.87)	<0.001	1.49 (1.21-1.84)	<0.001
Economic status: Poor and very poor	vs. Very good, good, and average	1.73 (1.59-1.89)	<0.001	2.01 (1.61-2.52)	<0.001
Education level: 9-12 years	vs. >12 years	1.13 (1.05-1.21)	<0.001	1.201 (1.003-1.437)	0.046
Health literacy^*^	per 1 point	0.87 (0.84-0.91)	<0.001	0.78 (0.70-0.86)	<0.001
Smoking: Current smoker	vs. Non- and ex-smokers	1.05 (0.96-1.14)	0.269	1.24 (1.03-1.49)	0.024
Alcohol consumption: Daily drinker	vs. Non- and occasional drinkers	0.85 (0.78-0.94)	<0.001	0.89 (0.73-1.09)	0.270
Physical activity: Absence	vs. Presence	1.45 (1.32-1.59)	<0.001	1.62 (1.28-2.04)	<0.001
Obesity: Presence	vs. Absence	1.24 (1.15-1.34)	<0.001	1.05 (0.87-1.27)	0.628
Annual visits to medical institutions	per 1 time/year	1.01 (1.01-1.01)	<0.001	1.009 (0.997-1.022)	0.140

Modified Poisson regression models were used to estimate the multivariable-adjusted RRs and 95% CIs for poor self-reported physical health (response variable: 1 = poor/very poor and 0 = very good/good/average) due to nonrestorative sleep. Age, sex, living alone, occupation, economic status, education level, health literacy, smoking, alcohol consumption, physical activity, obesity, and number of visits to medical institutions per year were included as covariates in the models. RR: risk ratio; CI: confidence interval. ^*^Communicative and Critical Health Literacy scale score.

Table 4 presents multivariable-adjusted RRs and 95% CIs for poor self-reported mental health due to nonrestorative sleep. Among the entire cohort, the adjusted RR for poor mental health was 2.21 (95% CI: 2.06-2.36; p < 0.001). Among participants without a history of present illness, the adjusted RR was 2.51 (95% CI: 2.18-2.89; p < 0.001). In both subgroups, nonrestorative sleep consistently yielded the highest RRs for poor mental health among all covariates. Moreover, in stratified models by regular-visit category, nonrestorative sleep remained associated with poor mental health across all categories (Table S4).

**Table 4.** Multivariable-Adjusted RRs and 95% CIs for Poor Self-Reported Mental Health due to Nonrestorative Sleep among Middle-Aged Adults in Japan.

Factors	Comparison	Entire cohort (n = 11,086)		Those without a history of present illness (n = 3,658)	
		Multivariable-adjusted RR (95% CI) for poor self-reported mental health	p-Value	Multivariable-adjusted RR (95% CI) for poor self-reported mental health	p-Value
Nonrestorative sleep	vs. Restorative sleep	2.21 (2.06-2.36)	<0.001	2.51 (2.18-2.89)	<0.001
Age	per 1 year	0.99 (0.98-0.99)	<0.001	0.988 (0.979-0.997)	0.010
Female	vs. Male	1.11 (1.04-1.19)	0.001	1.24 (1.08-1.42)	0.002
Living alone: Yes	vs. No	1.14 (1.06-1.24)	<0.001	1.33 (1.14-1.56)	<0.001
Occupation: Absence	vs. Presence	1.58 (1.48-1.69)	<0.001	1.50 (1.29-1.73)	<0.001
Economic status: Poor and very poor	vs. Very good, good, and average	2.04 (1.88-2.22)	<0.001	2.36 (1.98-2.81)	<0.001
Education level: 9-12 years	vs. >12 years	1.01 (0.95-1.08)	0.650	0.93 (0.82-1.06)	0.255
Health literacy^*^	per 1 point	0.84 (0.81-0.87)	<0.001	0.81 (0.75-0.87)	<0.001
Smoking: Current smoker	vs. Non- and ex-smokers	1.05 (0.98-1.14)	0.166	1.13 (0.98-1.30)	0.096
Alcohol consumption: Daily drinker	vs. Non- and occasional drinkers	0.90 (0.83-0.98)	0.012	0.91 (0.78-1.05)	0.206
Physical activity: Absence	vs. Presence	1.26 (1.16-1.36)	<0.001	1.03 (0.89-1.20)	0.662
Obesity: Presence	vs. Absence	1.00 (0.93-1.07)	0.917	0.94 (0.82-1.09)	0.440
Annual visits to medical institutions	per 1 time/year	1.005 (1.004-1.007)	<0.001	1.00 (0.99-1.02)	0.455

Modified Poisson regression models were used to estimate the multivariable-adjusted RRs and 95% CIs for poor self-reported mental health (response variable: 1 = poor/very poor and 0 = very good/good/average) due to nonrestorative sleep. Age, sex, living alone, occupation, economic status, education level, health literacy, smoking, alcohol consumption, physical activity, obesity, and number of visits to medical institutions per year were included as covariates in the models. RR: risk ratio; CI: confidence interval. ^*^Communicative and Critical Health Literacy scale score.

Missingness was limited across study variables (maximum 5.6% overall and 4.9% among variables included in the analytic models), with approximately 3% missingness for the outcomes and 0% for the exposure (Table S5). In sensitivity analyses using multiple imputation, nonrestorative sleep remained associated with poor self-reported physical and mental health. These associations were consistent across all subgroups: the entire sample, those without a history of present illness, and each regular-visit stratum (Tables S6-S9).

## Discussion

In this large-scale study, we demonstrated that nonrestorative sleep is strongly associated with poor self-reported physical and mental health among middle-aged adults in Japan. Greater proportions of poor health were consistently observed in the nonrestorative sleep group compared with the restorative sleep group in the entire cohort and among participants without a history of present illness. These associations remained significant after adjusting for a broad range of sociodemographic, lifestyle, and health-related factors, and were robust in sensitivity analyses stratified by regular-visit category.

The mechanisms linking nonrestorative sleep to poor health likely involve both biological and psychological processes. Prior research indicates that nonrestorative sleep is associated with heightened sympathetic activation before sleep, which disrupts sleep architecture and impairs restorative functions ^(30)^. Such disruption may lead to persistent fatigue, impaired cognition, and reduced daytime resilience, thereby influencing health perceptions. Psychological factors may also mediate this association. Heightened emotional reactivity, diminished coping capacity, and sustained negative affect have been linked to nonrestorative sleep and may exacerbate perceptions of poor health ^(31)^. Recent evidence further suggests that reduced resilience and increased stress sensitivity may contribute to this relationship ^(32)^. These biological and psychosocial mechanisms likely interact, reinforcing the persistence of poor self-reported physical and mental health.

Notably, the associations between nonrestorative sleep and poor health were consistent across the primary analytic groups (the entire cohort and participants without a history of present illness) and across sensitivity analyses stratified by regular-visit category. This suggests that nonrestorative sleep influences health perceptions regardless of existing disease burden. In the primary multivariable models, nonrestorative sleep showed strong, consistent associations with health perception compared with those observed for established predictors such as economic hardship ^(33), (34)^, low health literacy ^(35)^, and physical inactivity ^(36)^, highlighting its potential as a modifiable factor. From a nursing and public health standpoint, incorporating nonrestorative sleep into routine subjective health assessments may provide an efficient strategy for identifying individuals at risk who could benefit from supportive interventions, even in the absence of diagnosed conditions.

### Strengths and limitations

An important strength of this study is its large sample of middle-aged adults. However, this study also has several. First, the cross-sectional design precludes causal inference; prospective studies are needed to confirm these associations. Second, the response rate of 36.7% raises the possibility of selection bias, as individuals in relatively good health may have been more likely to participate. Third, the study was conducted in a single urban area, which may limit the generalizability of the findings to populations in other regions or settings. Fourth, nonrestorative sleep was assessed using a single self-report item designed for large-scale surveillance ^(13), (23)^. Although practical, this method may not capture the multidimensional nature of sleep; hence, validation with more comprehensive and psychometrically robust instruments is warranted. Finally, sleep duration and nocturnal insomnia symptoms (e.g., difficulty initiating or maintaining sleep) were not measured. Because nonrestorative sleep is closely associated with short sleep duration and nocturnal insomnia symptoms ^(37), (38)^, the observed associations with self-rated physical and mental health may partly reflect these unmeasured sleep dimensions (i.e., residual confounding). Future studies integrating qualitative and quantitative sleep indicators―including restoration, duration, and variability―are needed to clarify the relationship between sleep quality and subjective health in middle-aged adults.

## Conclusions

In this study, we found that nonrestorative sleep is strongly and consistently associated with poor self-reported physical and mental health among middle-aged adults in Japan. Individuals with nonrestorative sleep reported substantially higher proportions of poor health, regardless of illness status. Moreover, nonrestorative sleep remained an independent predictor of poor subjective health after adjustment for sociodemographic, behavioral, and health-related factors, yielding the highest RRs among all covariates in the primary multivariable models. These findings suggest that nonrestorative sleep may be a practical and modifiable indicator of health vulnerability in middle adulthood. Incorporating this measure into clinical and public health assessments could facilitate early identification of at-risk individuals and guide preventive interventions. Longitudinal studies using validated, multidimensional measures of nonrestorative sleep are needed to confirm causality and further elucidate its contribution to self-reported physical and mental health.

## Article Information

### Acknowledgments

We are grateful to all participants who took part in this research. We are grateful to all investigators and collaborators involved in this study.

### Author Contributions

Conceptualization, data curation, formal analysis, funding acquisition, investigation, project administration, literature review, and writing-original draft: Akiko Morimoto. Contributed to data interpretation, literature review, and critical review and editing of the manuscript: Hideaki Furuki, Aina Muta, and Liqian Sun. Conceptualization, data curation, funding acquisition, investigation, project administration, and critical review and editing of the manuscript: Nao Sonoda. All authors reviewed and approved the final version of the manuscript and agreed to be accountable for all aspects of the work.

### Conflicts of Interest

None

### Ethical Approval

The study protocol was written in accordance with the Declaration of Helsinki and was approved by the Institutional Review Boards of Osaka Prefecture University (date of approval 5 October 2020; approval no. 2020-28).

## Supplement

Supplementary Material
