# Supplementary material for: Nonrestorative Sleep and Poor Self-Reported Physical and Mental Health among Middle-Aged Japanese Adults: A Cross-Sectional Study
Source: JMA J. 2026 May 15;9(4):770–7. doi: 10.31662/jmaj.2025-0464 (PMC13453902; doi:10.31662/jmaj.2025-0464)
Supplement: Supplementary file 1 [file 2433-3298-9-4-0770-s001.pdf]

Table S1. Differences in Characteristics between Restorative Sleep and Nonrestorative Sleep Groups among Middle-Aged Adults in Japan by Regular-Visit Category.

Factors	Regular visits to non-psychiatric medical institutions only (n = 6,584)			Regular visits to psychiatric or psychosomatic institutions only (n = 309)			Regular visits to both non-psychiatric medical institutions and psychiatric or psychosomatic institutions (n = 535)		
	Restorative sleep	Nonrestorative sleep	p-value	Restorative sleep	Nonrestorative sleep	p-value	Restorative sleep	Nonrestorative sleep	p-value
n	3,854	2,730		201	108		291	244	
Age (years)	56.0 (6.7)	55.2 (6.5)	<0.001	51.3 (6.7)	51.0 (5.8)	0.634	53.0 (6.7)	53.6 (6.6)	0.232
Female, %	63.1	66.2	0.009	50.7	55.6	0.420	54.0	61.5	0.080
Living alone: Yes, %	14.3	13.6	0.432	19.9	15.7	0.369	17.5	18.4	0.783
Occupation: Absence, %	18.7	15.3	<0.001	53.7	46.3	0.213	48.5	44.7	0.383
Economic status: Poor and very poor, %	46.8	63.8	<0.001	61.7	76.9	0.007	65.3	73.8	0.034
Education level: 9-12 years, %	48.3	52.2	0.002	47.8	58.3	0.076	55.7	48.4	0.092
Health literacy* (points)	3.6 (0.7)	3.5 (0.7)	<0.001	3.1 (1.0)	3.1 (0.9)	0.820	3.3 (0.9)	3.3 (0.9)	0.678
Smoking: Current smoker, %	18.2	19.5	0.189	18.9	25.9	0.151	21.0	19.3	0.626
Alcohol consumption: Daily drinker, %	24.8	22.1	0.011	11.9	11.1	0.829	12.7	11.9	0.771
Physical activity: Absence, %	64.8	77.7	<0.001	76.6	83.3	0.167	73.9	81.6	0.035
Obesity: Presence, %	27.3	29.5	0.049	38.8	33.3	0.342	41.2	42.2	0.820
Annual visits to medical institutions (times/year)	8.0 (4.0, 12.0)	10.0 (5.0, 12.0)	<0.001	12.0 (6.0, 15.0)	12.0 (8.5, 20.0)	0.639	18.0 (12.0, 26.0)	20.0 (12.0, 30.0)	0.290

Age and health literacy were analyzed using t-tests and are shown as mean (standard deviation). Annual visits to medical institutions were analyzed with Mann-Whitney U tests and are shown as median (25th, 75th percentiles). Dichotomous and categorical data were analyzed with chi-squared tests and are presented as %.

*Communicative and Critical Health Literacy scale score.

Table S2. Differences in Self-Reported Physical and Mental Health between Restorative Sleep and Nonrestorative Sleep Groups among Middle-Aged Adults in Japan by Regular-Visit Category.

Factors	Regular visits to non-psychiatric medical institutions only (n = 6,584)			Regular visits to psychiatric or psychosomatic institutions only (n = 309)			Regular visits to both non-psychiatric medical institutions and psychiatric or psychosomatic institutions (n = 535)		
	Restorative sleep	Nonrestorative sleep	p-value	Restorative Sleep	Nonrestorative sleep	p-value	Restorative sleep	Nonrestorative Sleep	p-value
n	3,854	2,730		201	108		291	244	
Self-reported physical health, %			<0.001			<0.001			<0.001
Very good	14.0	3.2		9.5	4.6		9.3	2.5	
Good	26.5	14.1		17.4	8.3		15.8	4.9	
Average	44.8	48.4		49.3	36.1		37.5	30.7	
Poor	12.4	28.9		18.9	33.3		27.8	43.4	
Very poor	2.3	5.5		5.0	17.6		9.6	18.4	
Poor self-reported physical health*, %	14.7	34.3	<0.001	23.9	50.9	<0.001	37.5	61.9	<0.001
Self-reported mental health, %			<0.001			<0.001			<0.001
Very good	16.8	4.7		4.0	1.9		5.5	1.2	
Good	25.9	13.2		13.4	2.8		7.9	3.3	
Average	44.8	47.0		27.9	18.5		39.2	12.3	
Poor	11.0	28.6		34.8	33.3		35.7	48.0	
Very poor	1.5	6.6		19.9	43.5		11.7	35.2	
Poor self-reported mental health*, %	12.5	35.2	<0.001	54.7	76.9	<0.001	47.4	83.2	<0.001

Categorical data were analyzed with chi-squared tests and shown as %. Because percentages are rounded, the sum of the displayed percentages may not equal the displayed total for the corresponding category.
*Responses were dichotomized into: "very good/good/average" vs. "poor/very poor".

Table S3. Multivariable-Adjusted RRs and 95% CIs for Poor Self-Reported Physical Health due to Nonrestorative Sleep among Middle-Aged Adults in Japan by Regular-Visit Category.

Factors	Comparison	Regular visits to non-psychiatric medical institutions only (n = 6,584)		Regular visits to psychiatric or psychosomatic institutions only (n = 309)		Regular visits to both non-psychiatric medical institutions and psychiatric or psychosomatic institutions (n = 535)	
		Multivariable-adjusted RR (95% CI) for poor self-reported physical health	p-value	Multivariable-adjusted RR (95% CI) for poor self-reported physical health	p-value	Multivariable-adjusted RR (95% CI) for poor self-reported physical health	p-value
Nonrestorative sleep	vs. Restorative sleep	2.07 (1.89-2.27)	<0.001	1.81 (1.34-2.46)	<0.001	1.56 (1.31-1.85)	<0.001
Age	per 1 year	0.998 (0.992-1.005)	0.568	0.99 (0.96-1.01)	0.406	1.00 (0.99-1.02)	0.524
Female	vs. Male	0.92 (0.84-1.01)	0.075	1.00 (0.73-1.35)	0.981	1.14 (0.95-1.36)	0.154
Living alone: Yes	vs. No	1.116 (1.003-1.241)	0.045	1.03 (0.69-1.54)	0.876	1.06 (0.85-1.31)	0.604
Occupation: Absence	vs. Presence	1.73 (1.58-1.89)	<0.001	1.33 (0.97-1.84)	0.078	1.41 (1.18-1.68)	<0.001
Economic status: Poor and very poor	vs. Very good, good, and average	1.72 (1.55-1.90)	<0.001	2.07 (1.33-3.23)	0.001	1.41 (1.13-1.75)	0.002
Education level: 9-12 years	vs. >12 years	1.14 (1.05-1.25)	0.002	1.12 (0.83-1.52)	0.465	0.98 (0.82-1.16)	0.788
Health literacy*	per 1 point	0.88 (0.83-0.92)	<0.001	0.99 (0.85-1.14)	0.876	0.97 (0.88-1.06)	0.490
Smoking: Current smoker	vs. Non- and ex-smokers	0.97 (0.87-1.09)	0.619	1.25 (0.89-1.77)	0.193	1.13 (0.91-1.39)	0.265
Alcohol consumption: Daily drinker	vs. Non- and occasional drinkers	0.83 (0.74-0.93)	0.002	0.94 (0.56-1.58)	0.827	1.16 (0.90-1.49)	0.248
Physical activity: Absence	vs. Presence	1.39 (1.24-1.55)	<0.001	2.07 (1.17-3.66)	0.012	1.42 (1.09-1.83)	0.008
Obesity: Presence	vs. Absence	1.29 (1.18-1.40)	<0.001	0.78 (0.57-1.07)	0.125	0.98 (0.83-1.16)	0.805
Annual visits to medical institutions	per 1 time/year	1.008 (1.006-1.010)	<0.001	0.994 (0.986-1.001)	0.111	1.004 (1.002-1.005)	<0.001

Modified Poisson regression models were used to estimate the multivariable-adjusted RRs and 95% CIs for poor self-reported physical health (response variable: 1 = poor/very poor and 0 = very good/good/average) due to nonrestorative sleep. Age, sex, living alone, occupation, economic status, education level, health literacy, smoking, alcohol consumption, physical activity, obesity, and number of visits to medical institutions per year were included as covariates in the models.
RR: risk ratio; CI: confidence interval.
*Communicative and Critical Health Literacy scale score.

Table S4. Multivariable-Adjusted RRs and 95% CIs for Poor Self-Reported Mental Health due to Nonrestorative Sleep among Middle-Aged Adults in Japan by Regular-Visit Category.

Factors	Comparison	Regular visits to non-psychiatric medical institutions only (n = 6,584)		Regular visits to psychiatric or psychosomatic institutions only (n = 309)		Regular visits to both non-psychiatric medical institutions and psychiatric or psychosomatic institutions (n = 535)	
		Multivariable-adjusted RR (95% CI) for poor self-reported mental health	p-value	Multivariable-adjusted RR (95% CI) for poor self-reported mental health	p-value	Multivariable-adjusted RR (95% CI) for poor self-reported mental health	p-value
Nonrestorative sleep	vs. Restorative sleep	2.39 (2.17-2.64)	<0.001	1.28 (1.10-1.50)	0.002	1.70 (1.49-1.93)	<0.001
Age	per 1 year	0.99 (0.98-0.99)	<0.001	0.99 (0.98-1.01)	0.346	1.00 (0.99-1.01)	0.423
Female	vs. Male	1.11 (1.01-1.22)	0.029	0.96 (0.81-1.14)	0.649	0.93 (0.83-1.05)	0.273
Living alone: Yes	vs. No	1.116 (0.995-1.251)	0.060	0.92 (0.73-1.15)	0.454	0.93 (0.80-1.09)	0.369
Occupation: Absence	vs. Presence	1.44 (1.30-1.59)	<0.001	1.189 (0.999-1.415)	0.051	1.130 (1.002-1.275)	0.047
Economic status: Poor and very poor	vs. Very good, good, and average	1.99 (1.79-2.22)	<0.001	1.78 (1.37-2.31)	<0.001	1.52 (1.28-1.81)	<0.001
Education level: 9-12 years	vs. >12 years	1.08 (0.99-1.18)	0.095	1.04 (0.89-1.21)	0.659	0.98 (0.86-1.10)	0.683
Health literacy*	per 1 point	0.85 (0.80-0.89)	<0.001	0.97 (0.89-1.05)	0.419	0.99 (0.93-1.06)	0.773
Smoking: Current smoker	vs. Non- and ex-smokers	1.01 (0.91-1.13)	0.824	1.10 (0.92-1.32)	0.289	1.05 (0.91-1.22)	0.469
Alcohol consumption: Daily drinker	vs. Non- and occasional drinkers	0.94 (0.84-1.06)	0.326	1.11 (0.86-1.45)	0.426	1.04 (0.87-1.24)	0.678
Physical activity: Absence	vs. Presence	1.36 (1.22-1.52)	<0.001	1.25 (0.98-1.59)	0.071	1.17 (0.99-1.39)	0.069
Obesity: Presence	vs. Absence	0.94 (0.85-1.03)	0.183	1.05 (0.89-1.23)	0.556	0.96 (0.85-1.08)	0.501
Annual visits to medical institutions	per 1 time/year	1.004 (1.003-1.006)	<0.001	1.001 (0.999-1.002)	0.106	1.000 (0.998-1.002)	0.984

Modified Poisson regression models were used to estimate the multivariable-adjusted RRs and 95% CIs for poor self-reported mental health (response variable: 1 = poor/very poor and 0 = very good/good/average) due to nonrestorative sleep. Age, sex, living alone, occupation, economic status, education level, health literacy, smoking, alcohol consumption, physical activity, obesity, and number of visits to medical institutions per year were included as covariates in the models.
RR: risk ratio; CI: confidence interval.
*Communicative and Critical Health Literacy scale score.

Table S5. Missing Data Proportions across Study Variables.

Variable	n (%)
Self-reported Outcomes	
Physical health	375 (3.0)
Mental health	388 (3.1)
Exposure	
Sleep (restorative/nonrestorative)	0 (0.0)
Covariates	
Age	276 (2.2)
Sex	104 (0.8)
Living alone	0 (0.0)
Occupation	268 (2.2)
Economic status	232 (1.9)
Education level	403 (3.2)
Health literacy	615 (4.9)
Smoking	347 (2.8)
Alcohol consumption	376 (3.0)
Physical activity	0 (0.0)
Obesity	439 (3.5)
Annual visits to medical institutions	0 (0.0)
Auxiliary variables (imputation model only)	
Participation in health checkups	694 (5.6)
Daily working hours	0 (0.0)
Daily housework hours	0 (0.0)
Daily childcare hours	0 (0.0)
Daily caregiving hours	0 (0.0)
Health information source: Internet	0 (0.0)
Health information source: Healthcare providers	0 (0.0)
Health information source: Family/friends/coworkers	0 (0.0)
Stages of health behavior change	520 (4.2)

Values are numbers (% missing) among eligible participants (n = 12,446). Complete-case analyses included 11,086 participants.

Table S6. Multivariable-Adjusted RRs and 95% CIs for Poor Self-Reported Physical Health due to Nonrestorative Sleep among Middle-Aged Adults in Japan: Multiple Imputation Analysis.

Factors	Comparison	Entire cohort (n = 12,446)		Those without a history of present illness (n = 4,272)	
		Multivariable-adjusted RR (95% CI) for poor self-reported physical health	p-value	Multivariable-adjusted RR (95% CI) for poor self-reported physical health	p-value
Nonrestorative sleep	vs. Restorative sleep	2.08 (1.93-2.23)	<0.001	2.52 (2.12-3.00)	<0.001
Age	per 1 year	1.003 (0.998-1.008)	0.232	0.993 (0.981-1.005)	0.248
Female	vs. Male	0.96 (0.90-1.03)	0.294	0.87 (0.73-1.03)	0.102
Living alone: Yes	vs. No	1.14 (1.05-1.24)	0.003	1.07 (0.86-1.33)	0.524
Occupation: Absence	vs. Presence	1.68 (1.57-1.81)	<0.001	1.55 (1.30-1.86)	<0.001
Economic status: Poor and very poor	vs. Very good, good, and average	1.75 (1.62-1.90)	<0.001	2.04 (1.66-2.52)	<0.001
Education level: 9-12 years	vs. >12 years	1.12 (1.04-1.19)	0.002	1.182 (1.001-1.395)	0.049
Health literacy*	per 1 point	0.88 (0.85-0.92)	<0.001	0.79 (0.72-0.86)	<0.001
Smoking: Current smoker	vs. Non- and ex-smokers	1.03 (0.95-1.12)	0.462	1.18 (0.99-1.40)	0.069
Alcohol consumption: Daily drinker	vs. Non- and occasional drinkers	0.85 (0.77-0.93)	<0.001	0.89 (0.73-1.07)	0.217
Physical activity: Absence	vs. Presence	1.43 (1.31-1.56)	<0.001	1.52 (1.22-1.88)	<0.001
Obesity: Presence	vs. Absence	1.23 (1.15-1.32)	<0.001	1.08 (0.91-1.29)	0.387
Annual visits to medical institutions	per 1 time/year	1.008 (1.006-1.010)	<0.001	1.009 (0.997-1.021)	0.142

Modified Poisson regression models were used to estimate the multivariable-adjusted RRs and 95% CIs for poor self-reported physical health (response variable: 1 = poor/very poor and 0 = very good/good/average) due to nonrestorative sleep. Age, sex, living alone, occupation, economic status, education level, health literacy, smoking, alcohol consumption, physical activity, obesity, and number of visits to medical institutions per year were included as covariates in the models.
RR: risk ratio; CI: confidence interval.
*Communicative and Critical Health Literacy scale score.

Table S7. Multivariable-Adjusted RRs and 95% CIs for Poor Self-Reported Mental Health due to Nonrestorative Sleep among Middle-Aged Adults in Japan: Multiple Imputation Analysis.

Factors	Comparison	Entire cohort (n = 12,446)		Those without a history of present illness (n = 4,272)	
		Multivariable-adjusted RR (95% CI) for poor self-reported mental health	p-value	Multivariable-adjusted RR (95% CI) for poor self-reported mental health	p-value
Nonrestorative sleep	vs. Restorative sleep	2.16 (2.02-2.30)	<0.001	2.51 (2.19-2.87)	<0.001
Age	per 1 year	0.99 (0.98-0.99)	<0.001	0.988 (0.979-0.997)	0.008
Female	vs. Male	1.11 (1.04-1.18)	0.002	1.19 (1.05-1.35)	0.006
Living alone: Yes	vs. No	1.16 (1.08-1.25)	<0.001	1.35 (1.17-1.56)	<0.001
Occupation: Absence	vs. Presence	1.52 (1.42-1.61)	<0.001	1.42 (1.24-1.62)	<0.001
Economic status: Poor and very poor	vs. Very good, good, and average	2.06 (1.90-2.23)	<0.001	2.34 (1.98-2.77)	<0.001
Education level: 9-12 years	vs. >12 years	1.01 (0.95-1.07)	0.867	0.93 (0.82-1.05)	0.245
Health literacy*	per 1 point	0.84 (0.81-0.87)	<0.001	0.80 (0.75-0.86)	<0.001
Smoking: Current smoker	vs. Non- and ex-smokers	1.05 (0.98-1.13)	0.169	1.10 (0.95-1.26)	0.190
Alcohol consumption: Daily drinker	vs. Non- and occasional drinkers	0.89 (0.82-0.96)	0.003	0.89 (0.77-1.03)	0.107
Physical activity: Absence	vs. Presence	1.24 (1.15-1.33)	<0.001	1.01 (0.88-1.16)	0.902
Obesity: Presence	vs. Absence	1.01 (0.94-1.07)	0.840	0.96 (0.83-1.11)	0.572
Annual visits to medical institutions	per 1 time/year	1.006 (1.004-1.007)	<0.001	1.00 (0.99-1.02)	0.457

Modified Poisson regression models were used to estimate the multivariable-adjusted RRs and 95% CIs for poor self-reported mental health (response variable: 1 = poor/very poor and 0 = very good/good/average) due to nonrestorative sleep. Age, sex, living alone, occupation, economic status, education level, health literacy, smoking, alcohol consumption, physical activity, obesity, and number of visits to medical institutions per year were included as covariates in the models.

RR: risk ratio; CI: confidence interval.

*Communicative and Critical Health Literacy scale score.

Table S8. Multivariable-Adjusted RRs and 95% CIs for Poor Self-Reported Physical Health due to Nonrestorative Sleep among Middle-Aged Adults in Japan by Regular-Visit Category: Multiple Imputation Analysis.

Factors	Comparison	Regular visits to non-psychiatric medical institutions only (n = 7,185)		Regular visits to psychiatric or psychosomatic institutions only (n = 361)		Regular visits to both non-psychiatric medical institutions and psychiatric or psychosomatic institutions (n = 628)	
		Multivariable-adjusted RR (95% CI) for poor self-reported physical health	p-value	Multivariable-adjusted RR (95% CI) for poor self-reported physical health	p-value	Multivariable-adjusted RR (95% CI) for poor self-reported physical health	p-value
Nonrestorative sleep	vs. Restorative sleep	2.05 (1.88-2.23)	<0.001	1.62 (1.22-2.17)	<0.001	1.58 (1.34-1.86)	<0.001
Age	per 1 year	1.00 (0.99-1.01)	0.769	1.00 (0.97-1.02)	0.694	1.010 (0.998-1.022)	0.106
Female	vs. Male	0.91 (0.83-0.99)	0.033	0.94 (0.71-1.26)	0.688	1.14 (0.96-1.35)	0.146
Living alone: Yes	vs. No	1.14 (1.03-1.26)	0.013	1.09 (0.77-1.55)	0.624	1.02 (0.82-1.25)	0.875
Occupation: Absence	vs. Presence	1.69 (1.55-1.85)	<0.001	1.32 (0.97-1.79)	0.073	1.40 (1.18-1.66)	<0.001
Economic status: Poor and very poor	vs. Very good, good, and average	1.73 (1.57-1.90)	<0.001	1.86 (1.24-2.79)	0.003	1.54 (1.24-1.90)	<0.001
Education level: 9-12 years	vs. >12 years	1.13 (1.04-1.23)	0.003	1.10 (0.82-1.46)	0.534	0.95 (0.81-1.12)	0.561
Health literacy*	per 1 point	0.89 (0.85-0.94)	<0.001	1.00 (0.87-1.15)	0.989	0.96 (0.88-1.05)	0.349
Smoking: Current smoker	vs. Non- and ex-smokers	0.97 (0.87-1.08)	0.576	1.30 (0.94-1.79)	0.112	1.07 (0.87-1.31)	0.540
Alcohol consumption: Daily drinker	vs. Non- and occasional drinkers	0.83 (0.74-0.93)	0.001	0.91 (0.57-1.47)	0.710	1.12 (0.87-1.44)	0.371
Physical activity: Absence	vs. Presence	1.38 (1.24-1.53)	<0.001	2.23 (1.30-3.83)	0.004	1.40 (1.10-1.79)	0.006
Obesity: Presence	vs. Absence	1.28 (1.17-1.39)	<0.001	0.72 (0.52-0.99)	0.044	0.95 (0.81-1.11)	0.519
Annual visits to medical institutions	per 1 time/year	1.008 (1.006-1.009)	<0.001	0.995 (0.988-1.002)	0.134	1.004 (1.002-1.005)	<0.001

Modified Poisson regression models were used to estimate the multivariable-adjusted RRs and 95% CIs for poor self-reported physical health (response variable: 1 = poor/very poor and 0 = very good/good/average) due to nonrestorative sleep. Age, sex, living alone, occupation, economic status, education level, health literacy, smoking, alcohol consumption, physical activity, obesity, and number of visits to medical institutions per year were included as covariates in the models.
RR: risk ratio; CI: confidence interval.
*Communicative and Critical Health Literacy scale score.

Table S9. Multivariable-Adjusted RRs and 95% CIs for Poor Self-Reported Mental Health due to Nonrestorative Sleep among Middle-Aged Adults in Japan by Regular-Visit Category: Multiple Imputation Analysis.

Factors	Comparison	Regular visits to non-psychiatric medical institutions only (n = 7,185)		Regular visits to psychiatric or psychosomatic institutions only (n = 361)		Regular visits to both non-psychiatric medical institutions and psychiatric or psychosomatic institutions (n = 628)	
		Multivariable-adjusted RR (95% CI) for poor self-reported mental health	p-value	Multivariable-adjusted RR (95% CI) for poor self-reported mental health	p-value	Multivariable-adjusted RR (95% CI) for poor self-reported mental health	p-value
Nonrestorative sleep	vs. Restorative sleep	2.33 (2.12-2.56)	<0.001	1.25 (1.09-1.44)	0.002	1.70 (1.50-1.92)	<0.001
Age	per 1 year	0.99 (0.98-0.99)	<0.001	0.99 (0.98-1.01)	0.287	1.00 (0.99-1.01)	0.984
Female	vs. Male	1.12 (1.02-1.23)	0.016	0.95 (0.82-1.10)	0.471	0.94 (0.84-1.06)	0.306
Living alone: Yes	vs. No	1.120 (1.003-1.250)	0.044	0.97 (0.80-1.19)	0.782	0.90 (0.77-1.05)	0.191
Occupation: Absence	vs. Presence	1.41 (1.28-1.55)	<0.001	1.15 (0.98-1.35)	0.089	1.13 (1.01-1.27)	0.036
Economic status: Poor and very poor	vs. Very good, good, and average	2.03 (1.83-2.26)	<0.001	1.73 (1.36-2.19)	<0.001	1.55 (1.31-1.83)	<0.001
Education level: 9-12 years	vs. >12 years	1.06 (0.98-1.16)	0.164	1.02 (0.88-1.18)	0.777	0.99 (0.89-1.11)	0.872
Health literacy*	per 1 point	0.86 (0.81-0.90)	<0.001	0.95 (0.89-1.03)	0.197	1.00 (0.94-1.06)	0.973
Smoking: Current smoker	vs. Non- and ex-smokers	1.02 (0.92-1.14)	0.691	1.07 (0.90-1.26)	0.439	1.05 (0.92-1.20)	0.486
Alcohol consumption: Daily drinker	vs. Non- and occasional drinkers	0.95 (0.85-1.06)	0.370	1.05 (0.83-1.33)	0.690	0.97 (0.81-1.16)	0.713
Physical activity: Absence	vs. Presence	1.36 (1.22-1.52)	<0.001	1.22 (0.98-1.51)	0.071	1.18 (1.01-1.39)	0.040
Obesity: Presence	vs. Absence	0.96 (0.87-1.05)	0.372	0.99 (0.86-1.15)	0.944	0.93 (0.83-1.04)	0.196
Annual visits to medical institutions	per 1 time/year	1.004 (1.003-1.006)	<0.001	1.001 (0.999-1.002)	0.103	1.000 (0.998-1.002)	0.815

Modified Poisson regression models were used to estimate the multivariable-adjusted RRs and 95% CIs for poor self-reported mental health (response variable: 1 = poor/very poor and 0 = very good/good/average) due to nonrestorative sleep. Age, sex, living alone, occupation, economic status, education level, health literacy, smoking, alcohol consumption, physical activity, obesity, and number of visits to medical institutions per year were included as covariates in the models.
RR: risk ratio; CI: confidence interval.
*Communicative and Critical Health Literacy scale score.
